# Design of Infusion Schemes for Neuroreceptor Imaging: Application to [^11^C]Flumazenil-PET Steady-State Study

**DOI:** 10.1155/2016/9132840

**Published:** 2016-03-31

**Authors:** Ling Feng, Claus Svarer, Karine Madsen, Morten Ziebell, Agnete Dyssegaard, Anders Ettrup, Hanne Demant Hansen, Szabolcs Lehel, Stig Yndgaard, Olaf Bjarne Paulson, Gitte Moos Knudsen, Lars Hageman Pinborg

**Affiliations:** ^1^Neurobiology Research Unit, Copenhagen University Hospital, Rigshospitalet, Blegdamsvej 9, 2100 Copenhagen, Denmark; ^2^PET and Cyclotron Unit, Copenhagen University Hospital, Rigshospitalet, Blegdamsvej 9, 2100 Copenhagen, Denmark; ^3^Heart Centre, Aarhus University Hospital, Noerrebrogade 44, 8000 Aarhus, Denmark; ^4^Faculty of Health and Medicine Sciences, Copenhagen University, Blegdamsvej 3, 2200 Copenhagen, Denmark; ^5^Danish Research Centre Magnetic Resonance, Copenhagen University Hospital, Hvidovre Hospital, Kettegaard Allé 30, 2650 Hvidovre, Denmark; ^6^Epilepsy Clinic, Department of Neurology, Copenhagen University Hospital, Rigshospitalet, Blegdamsvej 9, 2100 Copenhagen, Denmark

## Abstract

This study aims at developing a simulation system that predicts the optimal study design for attaining tracer steady-state conditions in brain and blood rapidly. Tracer kinetics was determined from bolus studies and used to construct the system. Subsequently, the system was used to design inputs for bolus infusion (BI) or programmed infusion (PI) experiments. Steady-state quantitative measurements can be made with one short scan and venous blood samples. The GABA_A_ receptor ligand [^11^C]Flumazenil (FMZ) was chosen for this purpose, as it lacks a suitable reference region.* Methods*. Five bolus [^11^C]FMZ-PET scans were conducted, based on which population-based PI and BI schemes were designed and tested in five additional healthy subjects. The design of a PI was assisted by an offline feedback controller.* Results*. The system could reproduce the measurements in blood and brain. With PI, [^11^C]FMZ steady state was attained within 40 min, which was 8 min earlier than the optimal BI (B/I ratio = 55 min).* Conclusions*. The system can design both BI and PI schemes to attain steady state rapidly. For example, subjects can be [^11^C]FMZ-PET scanned after 40 min of tracer infusion for 40 min with venous sampling and a straight-forward quantification. This simulation toolbox is available for other PET-tracers.

## 1. Introduction

Among the kinetic modeling approaches to accurately quantify the neuroreceptor availability, tissue compartment modeling using arterial plasma as input is regarded as “gold standard.” However, the requirement of an arterial line is both invasive and less feasible in many clinical settings. Reference tissue modeling approaches use a brain region devoid of target neuroreceptors to obviate the arterial input function [1]. However, for numerous tracers a suitable anatomical reference region does not exist [2, 3]. As an alternative, the tracer can be given as a constant infusion and when steady-state conditions are attained, the patient can be positioned in the scanner [4]. At tracer steady state, venous instead of arterial blood may be used as a representation of the input function, since no arteriovenous difference exists when there is no net tissue uptake [4, 5]. However, the time to attain tracer steady state in brain and blood depends on the kinetics of the individual tracer and may in theory vary considerably from subject to subject. Thus, a preexperimental simulation system aiming at testing new tracers and guiding experimental procedures is important.

Here we describe a simulation system to model tracer uptake in plasma and brain regions based on the kinetics of tracers using the tracer administration as input; thus, it allows us to design the infusion scheme to attain fast steady-state condition. We illustrate the methodology and test our designed system using [^11^C]FMZ as an example. [^11^C]FMZ is a non-subtype-selective antagonist of the central benzodiazepine (BZR) site of the *γ*–aminobutyric acid (GABA_A_) receptor [6]. As a reference region, pons and white matter (WM) have been investigated, but both of them are susceptible to noise which may explain the failure of simplified reference tissue model (SRTM) to detect ipsilateral decreases in hippocampal nondisplaceable binding potential BP_ND_ in 3/15 patients with hippocampal sclerosis [7]. Different data-driven methods with and without arterial input function have been investigated for evaluating the feasibility of removing the invasive arterial line and shortening the scan time from 90 min to 60 min in [8]. Even though it was concluded that with 60 min of scan time the multilinear reference tissue model with fixed *k*_2_' (MRTM2) could provide useful estimation of regional BZR density, it gave an underestimation of BP_ND_ due to the fact that a contribution from blood volume was not taken into account. Image-derived input function has also been applied to quantify [^11^C]FMZ binding [9]; however, a number of arterial blood samples were still necessary. Thus, a steady-state approach for conducting quantitative [^11^C]FMZ-PET studies feasible in a clinical setting will be useful. This approach provides estimations of distribution volumes and is independent of assumptions for RTMs; thus, it is a better choice when no appropriate reference region is available. Even with an appropriate reference region at steady state, the binding potential can be estimated by simply taking the ratio *C*_*T*_/*C*_ND_ − 1, which is free of model assumptions.

## 2. Materials and Methods

### 2.1. Simulation Framework

The simulation system is based upon classical convolution analysis of systems in series. The design is based on the metabolite-corrected plasma input being convoluted with the impulse response function of the brain tissue to obtain brain time-activity curve (TAC) and is extended to model the plasma concentration by convoluting the tracer injection scheme with the impulse response function of the systemic circulation. The parameters in the simulation framework are estimated from the experimental data of dynamic scans and the system is assumed to be stationary and linear.

The simulation framework is divided into three parts.

Part A depicts the computation of the metabolite-corrected plasma concentration (*C*_*P*_) from the tracer administration scheme (*F*_Tracer_):

(1) ht=∑i=1naiexp⁡−bit,CPt=FTracert⊗ht,

where *h*(*t*) is the impulse response of the systemic circulation. A sum of three exponentials function was used for [^11^C]FMZ and *a*_*i*_ and *b*_*i*_ are the rate constants with unit min^−1^; ⊗ is the symbol of convolution. In the bolus experiments, the input was administrated as a constant infusion over 20 s; thus, *F*_Tracer_(*t*) is considered a step function with a duration of 20 s.

Part B depicts the computation of the total concentration (parent compound and radio-labeled metabolites) in whole-blood (*C*_*W*_) from *C*_*P*_:

(2) FParentt=c1exp⁡−d1t+c2exp⁡−d2t+c3,FP2Wt=f1−f2exp⁡−e1t,CWt=CPt/FParenttFP2Wt.

As shown, part B contains two functions: the parent fraction *F*_Parent_(*t*) and the plasma to whole-blood ratio *F*_*P*2*W*_(*t*). *C*_*P*_ is first converted to the total tracer concentration in plasma by the division by *F*_Parent_(*t*), and then the tracer concentration is further converted to *C*_*W*_ by the division by the plasma to whole-blood ratio *F*_*P*2*W*_(*t*). The form of these functions depends on the chemical and biological properties of the radioligand. For [^11^C]FMZ, exponential functions were chosen, and *c*_*i*_, *d*_*i*_, *e*_*i*_, and *f*_*i*_ are the parameters of the functions.

Part C is a tissue compartment model using *C*_*W*_ and *C*_*P*_ as inputs. The kinetics of [^11^C]FMZ can be described as a one-tissue compartment (1TC) model [10, 11]. However, the simulation system is designed for a two-tissue compartment model; thus, *k*_3_ and *k*_4_ were set to zero in this case:

(3) dC1tdt=K1CPt−k2C1t,CPETt=1−VBC1t+VBCWt,

where *K*_1_ is the delivery rate constant from plasma to brain tissue (*C*_1_); *k*_2_ is the washout rate constant from brain tissue; *C*_PET_ describes the concentration measured by the PET scanner, that is, radioactivity from both tissue and blood; *V*_*B*_ is the blood volume in brain tissue, which is fixed to 5%.

### 2.2. Programmed Infusion: A Feedback Control System

**Figure 1:**
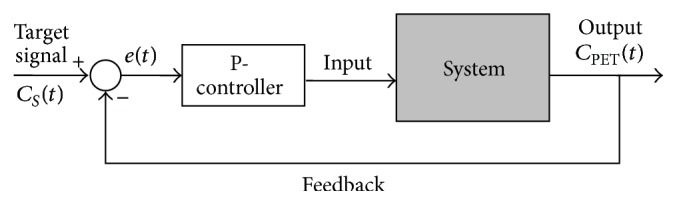
A feedback system with a proportional-controller. The P-controller adjusts the input to the system based on the difference between the target signal *C*_*S*_(*t*) and outcome of the system.

By using a proportional-controller (P-controller), an optimized PI scheme can be obtained. An offline linear feedback control system was developed, which designs the infusion protocol prior to PET experiments. The P-controller adjusts the input to the system proportionally to the difference between the target signal (*C*_*S*_(*t*)), which is the time-activity curve for a preselected region in a steady-state study, and the system output (*C*_PET_(*t*)), which is the simulated PET signal for the same region; see Figure 1. It can be described by

(4) et=CSt−CPETt,FTracert=Kpet,

where *K*_*p*_ is the proportional gain and it is set to 2.5 (determined by Simulink Control Design toolbox, Mathworks Inc.); *e*(*t*) is the instantaneous difference between the target signal *C*_*S*_(*t*) and the system output *C*_PET_(*t*). By providing a target signal, for example, the TAC of a receptor rich region attaining steady state, the feedback system adjusts the input *F*_Tracer_(·) repeatedly at a fix time interval, which is set to 30 s, to bring the system output similar to the target signal.

To design PI for [^11^C]FMZ, occipital cortex was chosen as the target region, as it is a BZR rich region and requires longer time to attain steady state in a BI setting. The target was created based on the kinetics of this region, and the TAC was maintained constant after reaching the peak induced by a bolus administration. We used the coupled optimization strategy, meaning the designed infusion scheme was also applied to other regions to ensure that the optimization does not cause a huge overshoot on the intermediate and low binding regions, which delays the attainment of steady state. In a classical feedback system, control signal can be negative, but as this is not the case for the infusion flow rate, it is fixed to zero instead.

### 2.3. Population-Based Infusion Scheme

The population-based system representation is built upon a number of bolus experiments to capture the tracer kinetics. To compare the results from different studies both the plasma TACs (Bq/mL) and the tissue TACs (Bq/mL) are normalized by injected dose per bodyweight unit (kBq/kg), resulting in standardized uptake values (SUVs) (g/mL). The three parts of the simulation framework were fitted to the individual bolus data. Mean values of the subject-specific parameters across all bolus experiments were used for population-based simulations.

### 2.4. Human Studies

Sixteen PET scans were conducted in ten healthy subjects (3 men; mean age ± SD: 26 ± 8 years, range 20–46 years). The study was approved by the Regional Ethics Committee (KF 01280377) and informed consent was obtained from all subjects. All subjects had no history of neurological or psychiatric disorders. Physical examinations, ECG, and routine blood test were normal in all subjects.

The protocols carried out for each subject are listed in Table 1. Five bolus dynamic PET scans were conducted to adapt the kinetics of [^11^C]FMZ to the simulation system. To verify that the simulation system can represent the dynamic and physiological characteristics of [^11^C]FMZ, one of the subjects was studied twice. First, a bolus alone experiment was done and based upon the kinetic parameters determined from this bolus study the optimal PI scheme was determined for this subject. Next, a PI experiment was conducted in the same subject. Finally, based on the population-based simulation, five pairs of PI and BI experiments were done in five new subjects.

### 2.5. Radiochemistry and PET Image Acquisition

Flumazenil was labeled with carbon-11 using methyl triflate [12], which was prepared online from [^11^C]methyl iodide [13]. Radiochemical purity of [^11^C]FMZ was greater than 99%. At the time of injection, the specific radioactivity was 279.7 GBq/*μ*mol (range, 23.3–711 GBq/*μ*mol; *n* = 16); the injected radioactivity was 340.7 MBq (range, 46.4–699 MBq; *n* = 16); and the total radioactivity of the double-scanned subjects received was 379.0 MBq (range, 118.7–733 MBq; *n* = 6). The average injected mass was 1.34 *μ*g (range, 0.03–8.48 *μ*g; *n* = 16). The BI and PI experiments were performed using a computer controlled infusion pump (Harvard PHD 2000 Infusion pump).

The high-resolution research tomography (HRRT) scanner (Siemens AG), running in list-mode, was used for acquisition of PET data. Emission scans started simultaneously with the injection/infusion of tracer. Data were recorded in 3-dimensional mode for 90 min for bolus studies and 120 min for BI and PI studies. For the dynamic scans, PET data were reconstructed into 35 dynamic frames of increasing length (6 × 5, 10 × 15, 4 × 30, 5 × 120, 5 × 300, and 5 × 600 s) using the Ordered Subset Expectation Maximization iterative method; and for the longer scans 3 × 600 s frames were added. Images consist of 207 planes of 256 × 256 voxels with the size of 1.22 × 1.22 × 1.22 mm.

Structural brain imaging (T1- and T2-weighted) of magnetic resonance imaging (MRI) was performed with a 3.0 T Trio scanner (Siemens Medical Solutions, Erlangen, Germany) with a voxel size of 1 × 1 × 1 mm.

### 2.6. Input Measurement

In the infusion experiments, cannulas were inserted into both cubital veins for radiotracer administration and blood sampling, respectively. In the bolus studies, a cannula was inserted into the radial artery of the nondominant arm for arterial blood sampling. During the first 10 min of scanning, the coincidences were counted continuously in the whole-blood with an ABSS autosampler (Allogg Technology) and three samples were drawn manually to rectify the autosamples. To determine the plasma to whole-blood ratio, blood samples were drawn manually at 18 time points with increasing time intervals. They were measured in a well counter (Cobra 5003; Packard Instruments) and data were decay-corrected to the time of injection. In addition, nine blood samples were drawn during scanning for metabolite analysis by radio-high performance liquid chromatography (HPLC). All the equipment was cross-calibrated to the HRRT scanner. The percentage of parent compound was fitted using *F*_Parent_(*t*). The metabolite-corrected plasma input function was calculated as the product of this biexponential function and the total plasma concentration.

### 2.7. Data Analysis

PET images were inspected for movements, using a set of in-house MATLAB based functions and an AIR 5.2.5 algorithm [14]: each dynamic frame was filtered by a Gaussian 3D filter (FWHM 10 mm) and thresholded to only include brain voxels. Thereafter, the rigid transformation was estimated for the frames with length greater than 15 s to the first 5 min frame, which has less noise than other frames. For all images, the detected median voxel movement was less than the precision of the algorithm that is 3 mm. Thus, no movement correction was needed.

Applying the coregistration algorithm implemented in Statistical Parametric Mapping (SPM) [15], the MRI image was coregistered to the mean PET image of the same subject from time 75 s to 20 min after the start of scanning, as within this time interval tracer distribution better reflects the cerebral perfusion. An automatic delineation of VOIs was performed using probability maps [16]. The TACs of nine VOIs were then calculated. Radioactivity in regions located in both hemispheres was calculated as the weighted average of radioactive concentrations in left and right sides weighted with the VOI volume. Finally, each tissue TAC was normalized by the injected dose per bodyweight unit of the subject, yielding a regional SUV.

Bolus data were fitted to a 1TC model. The Akaike Information Criterion (AIC) was used to determine the number of exponentials in modeling arterial plasma curve (*C*_*P*_) that is part A of the simulation system. In general, we consider that brain tissue attains steady-state condition when the individual measurements in the time-activity curve are within 5% of the plateau. Due to the noise in the data, the plateau was determined by averaging the five last time-activity values in order to get a robust estimate. To investigate if both blood and brain tissues attained steady state, the time development of distribution volume *V*_*T*_ was studied for measuring the time of attaining steady-state conditions. The distribution volume was calculated as *V*_*T*_ = *C*_*T*_/*C*_*P*_ = (*C*_PET_ − *V*_*B*_ *C*_*W*_)/(1 − *V*_*B*_)/*C*_*P*_. The coefficient of variance (COV) of the mean *V*_*T*_ was used to measure the robustness of BI and PI across five infusion subjects. COV is the standard deviation of the measurements across subjects divided by the mean.

PMOD software (version 3.0; PMOD Technologies Inc.) was used for kinetic modeling. Data preprocessing and function fitting were done in MATLAB, so was the statistical analysis: paired-sample *t*-test and Pearson's linear correlation; and the simulation system was designed in Simulink (Simulink version 7.5; MATLAB version 7.10; Mathworks Inc.).

## 3. Results

### 3.1. Bolus Studies

The TACs of metabolite-corrected plasma from the 5 bolus studies were fitted to part A (1) separately. The lowest AIC values suggested a sum of three exponentials. The microparameters are given in Table 2. The variations of the microparameters over 5 studies were pronounced, which may be due to modeling uncertainty of the microparameters and interindividual variability. However, the macroparameters *a*_*i*_/*b*_*i*_ were much more stable and comparable across subjects. The results of kinetic modeling are presented in Table 3.

### 3.2. Bolus Infusion Studies

For population-based bolus infusion studies, the system was built based on averaged kinetic parameters from the bolus studies. A B/I ratio of 55 min generated the fastest steady-state conditions across receptor rich, medium, and poor regions in the simulation.

**Figure 2:**
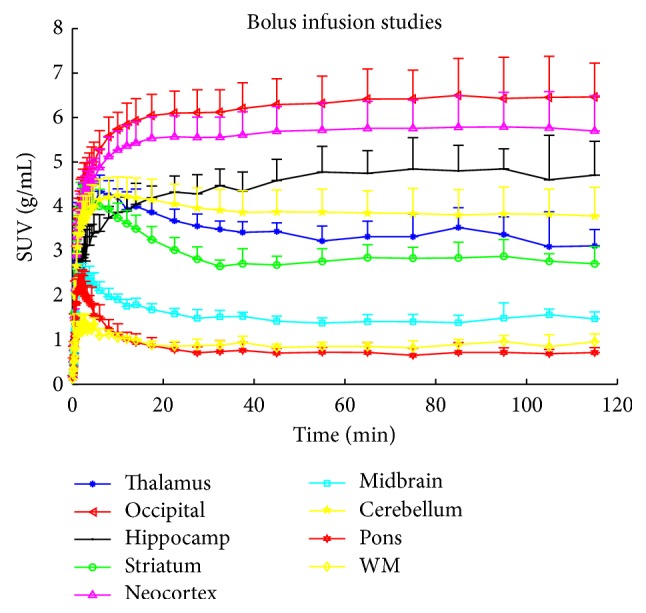
Bolus infusion studies with a bolus infusion ratio B/I = 55 min (*n* = 5): mean and standard deviation (one direction) of the standard uptake volume in occipital cortex, neocortex, hippocampus, cerebellum, thalamus, pallidostriatum, midbrain, white matter, and pons.

Five PET studies with a B/I ratio of 55 min were then conducted in five new subjects.  Figure 2 shows the mean SUVs and the standard deviation for nine regions. The B/I protocol of 55 min did bring the brain tissues to steady-state condition, at which point the venous tracer concentration equals the arterial concentration. For the first 80 min, the HPLC measurements were quite reliable, but for the later samples the variation of the metabolite measurement increased as a function of lower counts. Thus, the distribution volume as a function of time *V*_*T*_(*t*) was only calculated from the beginning of tracer infusion to 80 min. The plateau for steady-state estimation was calculated as an average of *V*_*T*_(*t*) from time of 35 min (frame 30) to 80 min (frame 34) after tracer administration. During one BI scan the HPLC analysis failed and thus Table 4 gives the averaged time of steady-state attainment across 4 BI studies. The mean and standard deviation of the averaged *V*_*T*_ from 35 to 80 min across 4 subjects are given in Table 3.

### 3.3. Programmed Infusion Studies

**Figure 3:**
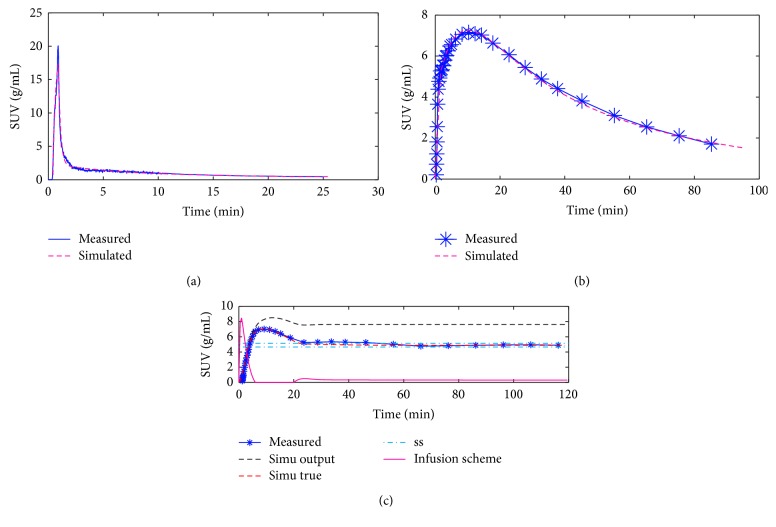
The standardized uptake values (SUVs) of a bolus and a programmed infusion (PI) study on the same subject. (a) and (b) show arterial plasma SUV and occipital cortex SUV of a bolus study, respectively. From the bolus data, a PI scheme was designed. (c) shows the infusion scheme; the measured occipital cortex SUV; the simulated occipital cortex SUV “simu output” with *k*_2_ = 0.06 min^−1^, model parameter from the bolus study; the estimated SUV “simu true” with *k*_2_ = 0.096 min^−1^, suggested by the measured PI data; and the definition of steady state “ss”, which is ±5% of the plateau. The infusion scheme is unitless, and it shows the relative development of infusion with time.

A subject-specific PI study was carried out in one subject to verify the simulation system. This subject-specific PI scheme was determined by the simulation system using the kinetic parameters from the bolus study of the same subject. The subject-specific PI scheme designed through a P-controller feedback system is given in Figure 3(c). Most prominently the pump was stopped for 14 min after the initial 6 min of infusion and thereafter a slow infusion took place with flow rates updated every 30 s. The simulation system could reproduce the SUVs for the metabolite-corrected plasma and the brain tissue (Figures 3(a) and 3(b)), and the designed PI scheme brought the brain tissue into steady state (Figure 3(c)).

**Figure 4:**
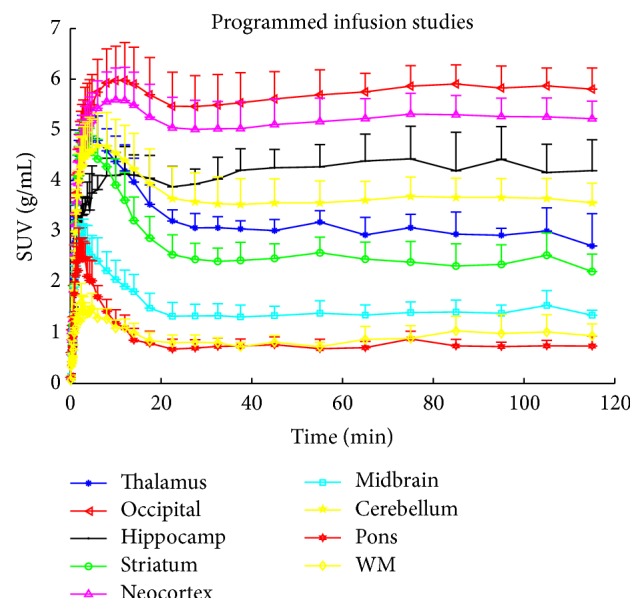
Programmed infusion: mean and standard deviation (one direction) of the standard uptake volume in occipital cortex, neocortex, hippocampus, cerebellum, thalamus, pallidostriatum, midbrain, white matter, and pons (*n* = 5).

The population-based PI scheme was designed using the averaged kinetic parameters across five bolus studies. The scheme suggested an initial infusion of 4 min and a pause of 17 min before restarting infusion. SUVs of 9 brain regions are shown in Figure 4. Table 4 gives the averaged time of steady-state attainment across 5 PI scans measured by the time development of *V*_*T*_. The experimental results showed that the PI scheme brought the blood and brain tissues to steady-state condition earlier than the BI scheme (*p* = 0.0048); across the brain regions, the time shortened by the PI scheme was 8 min on average. The COV of the mean *V*_*T*_ from 35 to 80 min of each scan in different brain regions was calculated. No significant difference between BI and PI of the estimated *V*_*T*_ was found (*p* = 0.712). The mean and standard deviation of *V*_*T*_ averaged from 35 to 80 min after the beginning of the PI studies (*n* = 5) are given in Table 3. The mean *V*_*T*_ values across four BI studies correlated significantly with the mean *V*_*T*_ values across five PI studies (*r* = 0.9996, *p* = 4.03*e* − 12). Furthermore, they also correlated with the mean *V*_*T*_ values from 1TC modeling of 5 bolus studies: *r* = 0.9991, *p* = 6.52*e* − 11 (BI versus 1TC); and *r* = 0.9996, *p* = 3.70*e* − 12 (PI versus 1TC). The infusion studies overestimated the *V*_*T*_ by 10.5% (BI) and 16.5% (PI) compared to 1TC.

## 4. Discussion

The purpose of this study was to introduce a simulation system that utilizes the known tracer kinetics from bolus studies to apply it to the design of PI and BI experiments in humans. The concept of our simulation system is similar to the UIPump-system which was tested in Sprague-Dawley rats for microPET scanning [17]. The UIPump-system is feasible to small animal studies, where the tracer kinetics is faster than in humans. However, for human PET a more complex system is necessary. In contrast to the UIPump-system approach which simulates how the rodent body handles an exposure of a radioligand with one impulse response function, we divided the process into three parts: part A simulates the metabolite-corrected plasma concentration using the infusion paradigm; part B prepares the total tracer concentration in whole-blood using the output from part A; then, part C simulates the tissue TACs using blood TACs as inputs. The simulation output from each part was verified with experimental data.

With the proposed simulation system, an infusion scheme can be designed to replace arterial blood sampling by a number of venous samples for quantitative estimation of tracer binding. Although taking only a single blood sample has been proposed [18], we recommend taking several venous blood samples. Firstly, it is to ascertain the steady-state attainment and, secondly, to increase the precision of the measure without increasing the invasiveness, given that a venous line is already in place.

### 4.1. The Simulation System

The occipital cortex is the VOI with the highest [^11^C]FMZ binding (Table 3) and accordingly, following a constant infusion of [^11^C]FMZ, steady state will be attained later in this VOI compared to other VOIs with a lower binding. Thus, we chose the occipital cortex as the target region to design a PI scheme with the feedback controller. By optimizing the infusion scheme for the VOI with the highest binding potential, the TACs of the low binding VOIs will initially overshoot, as illustrated by the experimental results (Figure 4). The P-controlled feedback system suggested a negative input after the initial infusion to attain the fastest steady-state conditions. Thus, a nonnegative constraint was applied when designing the infusion scheme, which subsequently introduced a pause during the course of infusion. The drop in the high binding regions (i.e., 10–25 min) was mainly due to the pause.

To validate the simulation system, subject 5 was studied twice: first, a bolus study was done, based upon which the optimal PI scheme was determined; then, a PI experiment was conducted. The infusion scheme and the experimental and simulated SUVs are shown in Figure 3. The comparison of the simulated and the experimental data implies that the simulation system could represent the outcomes from the [^11^C]FMZ experiments. The difference between the simulated output and the measured SUV is probably related to the uncertainty in the estimation of the compartment model parameters from the bolus experiment and interexperiment variation from the bolus to the PI experiment. Based on the behavior of the simulated SUV, the *k*_2_ = 0.060 min^−1^ estimated from the bolus data was too low compared to the actual *k*_2_ of the subject in the PI experiment. When increasing the *k*_2_ to 0.096 min^−1^ and keeping *K*_1_ unchanged, the resulting estimation aligned with the measured SUV much better.

### 4.2. The PET Experiments

The averaged *V*_*T*_ curve across five PI experiments attained steady state within 40 min for high and intermediate binding regions. Identification of steady-state condition was challenging, particularly in receptor poor regions, where the binding is more sensitive to noise, especially at the latter frames when count statistics is low. There is no good way to reduce noise in TACs except to inject a higher radioactivity dose or to confine binding assessment to larger brain regions only. The same applies to the plasma measurements, where the low count statistics reduces the accuracy of the measurements, especially in the HPLC measurements.

A limitation of BI studies and PI studies is that they require use of population-based infusion schemes. The interindividual variability in terminal plasma clearance is clearly demonstrated in Table 2 and different subjects attained tracer steady state at slightly different times in different VOIs (Table 4). Individual variation in time to attain tracer steady state using a population-based infusion scheme is reduced by waiting for data acquisition (PET scan and blood sampling) until the majority of subjects are expected to have attained steady state in blood and brain and by dividing the acquisition time into 5–10 min frames for post hoc verification of tracer steady state and possible exclusion of frames. Compared to the population-based BI scheme, the PI scheme hastened the steady-state attainment by 8 min on average, when looking at the time development of the distribution volume. Whereas this difference may not sound all that impressive, for carbon-11 labeled tracers, it still means gaining 24% of radioactivity. In addition, the difference may be larger for other radioligands. Compared to BI scheme which is determined by only one parameter, the B/I ratio, PI enables more sophisticated input patterns based on both the tracer kinetics in plasma and brain tissues, which leads to a faster steady-state condition attainment.

## 5. Conclusion

We here, by example of [^11^C]FMZ, introduce a simulation system that reproduces both the blood and brain time-activity curves from PET experiments. Thus, the system can be used to design infusion schemes, both BI and PI, to bring both the blood and brain into steady state as early as possible. As expected, the PI scheme attained steady-state conditions faster than the optimal BI scheme. We recommend starting [^11^C]FMZ-PET scanning at 40 min after the beginning of tracer administration and scan for 40 min with, for example, 5 venous samples and a straight-forward computation of the distribution volume.

## Figures and Tables

**Table 1:** List of subjects and protocols involved in this study.

	Bolus	Bolus infusion	Programmed infusion
Subj 1	X		
Subj 2	X		
Subj 3	X		
Subj 4	X		
Subj 5	X		Subject-specific
Subj 6		B/I = 55 min	Population-based
Subj 7		B/I = 55 min	Population-based
Subj 8		B/I = 55 min	Population-based
Subj 9		B/I = 55 min	Population-based
Subj 10		B/I = 55 min	Population-based

Note: B/I is the bolus infusion ratio.

**Table 2:** Metabolite-corrected plasma time-activity curve fitting.

	*a*_1_ (min^−1^)	*b*_1_ (min^−1^)	*a*_1_/*b*_1_	*a*_2_ (min^−1^)	*b*_2_ (min^−1^)	*a*_2_/*b*_2_	*a*_3_ (min^−1^)	*b*_3_ (min^−1^)	*a*_3_/*b*_3_
Bolus-1	2.503	2.579	0.97	0.109	0.107	1.02	0.045	0.010	4.56
Bolus-2	0.394	0.765	0.52	5.125	6.302	0.81	0.079	0.022	3.61
Bolus-3	0.455	0.860	0.53	6.353	7.990	0.80	0.103	0.029	3.58
Bolus-4	4.233	5.103	0.83	0.157	0.134	1.17	0.065	0.017	3.89
Bolus-5	6.544	7.142	0.92	0.189	0.178	1.06	0.086	0.018	4.79

Mean ± SD			0.75 ± 0.22			0.97 ± 0.16			4.09 ± 0.56

Note: *a*_*i*_ and *b*_*i*_ are the rate constants of the impulse response function to represent the concentration of parent radioligand in arterial plasma; see (1).

**Table 3:** Model parameters of one-tissue compartment model (1TC) and steady-state approaches: programmed infusion (PI) and bolus infusion (BI).

	1TC			PI	BI
	*K*_1_ (mL min^−1^ mL^−1^)	*k*_2_ (min^−1^)	*V*_*T*_	*V*_*T*_	*V*_*T*_
Occipital cortex	0.43 ± 0.07	0.05 ± 0.01	8.05 ± 0.56	9.00 ± 1.56	8.89 ± 1.73
Neocortex	0.41 ± 0.06	0.06 ± 0.01	7.36 ± 0.46	8.16 ± 1.30	7.97 ± 1.60
Hippocampus	0.28 ± 0.04	0.05 ± 0.01	6.11 ± 0.49	6.79 ± 1.44	6.57 ± 1.42
Cerebellum	0.41 ± 0.06	0.08 ± 0.01	4.97 ± 0.70	5.62 ± 0.85	5.39 ± 1.12
Thalamus	0.44 ± 0.06	0.11 ± 0.01	4.13 ± 0.38	4.83 ± 1.11	4.66 ± 0.93
Pallidostriatum	0.45 ± 0.07	0.13 ± 0.02	3.35 ± 0.34	3.86 ± 0.93	3.83 ± 0.75
Midbrain	0.25 ± 0.04	0.16 ± 0.01	1.59 ± 0.17	2.05 ± 0.31	1.92 ± 0.28
White matter	0.11 ± 0.02	0.12 ± 0.01	0.93 ± 0.08	1.12 ± 0.13	1.11 ± 0.20
Pons	0.31 ± 0.06	0.33 ± 0.04	0.91 ± 0.09	1.10 ± 0.15	0.85 ± 0.07

The mean and standard deviation are reported. *V*_*T*_ is defined as *K*_1_/*k*_2_ for 1TC and *C*_*T*_/*C*_*P*_ for PI and BI averaging from 35 to 80 min after the beginning of tracer administration.

**Table 4:** Time for the distribution volume attaining steady-state conditions for population-based programmed infusion (PI) and bolus infusion (BI).

	Time (min)	
	PI	BI
Occipital cortex	**25** ± 12	38 ± 14
Neocortex	**20** ± 9	35 ± 11
Hippocampus	34 ± 10	**29** ± 9
Cerebellum	**27** ± 14	39 ± 10
Thalamus	**30** ± 14	42 ± 9
Pallidostriatum	**40** ± 14	41 ± 0
Midbrain	**27** ± 14	36 ± 6
White matter	**45** ± 12	53 ± 9
Pons	**44** ± 15	53 ± 9

The mean and standard deviation are reported. The steady state was determined as the time point, when the change of the time-activity curve was within ±5% of the plateau.

## Abbreviations

BI: Bolus infusion
PI: Programmed infusion
FMZ: Flumazenil
SRTM: Simplified reference tissue model
TAC: time-activity curves
1TC: One-tissue compartment model
P-controller: Proportional-controller
SUV: Standardized uptake value
HRRT: High-resolution research tomography
COV: Coefficient of variance
VOI: Volume of interest.
